# Tannic Acid Shaped Microbiome Composition in Midguts and Rearing Microcosms of *Aedes triseriatus* (Say)

**DOI:** 10.21203/rs.3.rs-7706154/v1

**Published:** 2025-10-27

**Authors:** Shicheng Chen, Liang Cui, Bin Zuo, Jiangchao Zhao, Edward D. Walker

**Affiliations:** 1. Medical Laboratory Science, College of Health and Human Sciences, Northern Illinois University, Dekalb, IL, USA.; 2. State Key Laboratory Breeding Base of Marine Genetic Resources, Third Institute of Oceanography, MNR, Xiamen, PR China.; 3. Guangdong Laboratory for Lingnan Modern Agriculture, Animal Functional Microbiome Lab, College of Animal Science, South China Agricultural University, Guangzhou, China; 4. Department of Microbiology, Molecular Genetics and Immunology, Michigan State University, East Lansing, MI 48824 USA

**Keywords:** *Aedes triseriatus*, comparative microbiome analysis, tannic acid, diversity and function prediction

## Abstract

Tannic acid (TA), a polyphenol derived from plants, often accumulates in water-holding containers where mosquitoes develop. Yet, its effects on mosquito gut microbiota remain poorly understood, representing an important knowledge gap. Because mosquito-associated microbiota are vital for host development, nutrition, and immunity, uncovering how TA shapes these microbial communities may yield new insights into mosquito biology and vector control strategies. In this study, we conducted a comparative analysis of bacterial communities in *Aedes triseriatus* midguts and rearing microcosms with or without TA supplement. Addition of TA at 0.35 mg/mL caused up to 50% larval *Ae. triseriatus* mortality, whereas combined supplementation with TA and kanamycin (100 μg/mL) increased mortality to 75% relative to controls. TA treatment significantly reduced microbial Chao 1 richness and Shannon diversity in larval and adult mosquito guts, while water and leaf samples were not affected. Distinct microbial community structures were observed between TA-treated and control groups across larvae, adults, water, and leaf surfaces. Pseudomonadota and Bacteroidota dominated all samples, with TA increasing the relative abundance of Pseudomonadota while decreasing Bacteroidota. Notably, *Pseudomonas* was enriched in TA-treated water, leaf surfaces, and larval midguts, suggesting a role in tannic acid detoxification. PICRUSt functional predictions indicated enrichment of carbohydrate and amino acid metabolism and membrane transport pathways under TA exposure, reflecting adaptive microbial responses to TA stress. Our findings highlighted how TA shapes mosquito microbiota and habitat quality, offering potential avenues to manipulate microbial communities as a biocontrol strategy for mosquito larvae.

## Introduction

*Aedes triseriatus* (Say) (Diptera: Culicidae), the primary vector of La Crosse virus in the Midwestern United States, breeds in natural habitats such as water-filled tree holes, as well as artificial containers like discarded tires [1]. These microhabitats are sustained by the accumulation of senescent leaves, plant detritus, and dead invertebrates, which drive microbial activity and support the base of the aquatic food web [2,3]. Although the water in these habitats is regularly replenished by rain water moving into tree holes as stemflow [2], the decomposition of leaf litter through chemical and biological processes alters its nutritional quality for mosquito larvae and other aquatic invertebrates [2]. Microbial metabolism in particular modifies water chemistry and releases various compounds, including tannic acid, which can accumulate to toxic levels and negatively impact mosquito larval development [4]. Despite its ecological relevance, the physiological and behavioral effects of leaf-derived compounds such as tannic acid on mosquito larvae remain poorly understood. Tannins in tree hole water inhibited growth of the western tree hole mosquito, *Ae. sierrensis* and caused larval mortality in a dose dependent manner [5,6]. Histological analyses have shown that midgut epithelium was damaged by TA or its derives, causing cellular degeneration - particularly affecting the clear cells of the anterior midgut before the dark cells of the posterior region [7]. Additionally, TA has been reported to interfere with the digestion of starches, lipids, and proteins, thereby disrupting nutrient absorption and leading to growth inhibition, as observed in *Hyphantria cunea* larvae [8].

Tannic acid (TA), a plant-derived polyphenol, exhibits broad-spectrum antimicrobial and anti-biofilm activities against various bacteria and fungi *in vitro*. It inhibited growth of both Gram-positive and Gram-negative bacteria by disrupting cell membranes, chelating essential metal ions, and interfering with enzymatic functions [9,10]. Tannic acid also effectively prevented biofilm formation by reducing microbial adhesion to surfaces, disrupting cell-to-cell signaling, and destabilizing extracellular polymeric substances [11,12]. These properties were observed in diverse microbial taxa, including *Streptococcus*, *Bacillus*, and fungal genera such as *Candida* [13–16], underscoring its ecological significance and potential applications in microbial community modulation and biofilm management [17]. *In vivo* studies in insects demonstrated that tannic acid disrupted gut microbial communities and interfere with digestive processes, contributing to reduced growth and survival [18]. For example, in *Hyphantria cunea* larvae, tannic acid ingestion caused significant damage to midgut epithelial cells and impaired the activities of key digestive enzymes, including amylase, lipase, and protease, resulting in poor nutrient absorption and growth retardation [8,18]. Additionally, tannic acid was shown to increase gut permeability in insects, potentially facilitating microbial translocation and immune activation [19]. These findings highlighted the dual role of tannic acid in directly affecting insect physiology and indirectly influencing development through microbiota modulation [18]. Given that mosquito larvae depended heavily on their gut microbiota for digestion and development, elevated levels of tannic acid possibly disrupted these microbial communities (either within the gut or the larval habitat), thus impeding larval development [20]. Furthermore, midgut epithelial damage may compromise the gut barrier, allowing microbial translocation into the hemocoel and potentially leading to sepsis. This hypothesis is supported by studies showing enhanced larvicidal efficacy of *Bacillus thuringiensis* when co-administered with tannic acid [21].

Several bacteria possess the ability to detoxify tannic acid through enzymatic degradation pathways, enabling them to survive in tannin-rich environments such as leaf litter or herbivore insect guts [22,23]. Key enzymes include tannase, which hydrolyzes tannic acid into gallic acid and glucose. Gallate decarboxylase then further metabolizes gallic acid [24]. Microbes such as *Lactobacillus plantarum*, *Bacillus subtilis*, *Enterococcus faecalis*, and *Streptococcus gallolyticus* have been shown to express these enzymes and tolerate tannic acid exposure [25–28]. This microbial detoxification not only benefits the bacteria themselves but may also alleviate tannin toxicity for host organisms, such as insects, by reducing oxidative stress and supporting gut homeostasis [17,18,22,24]. In this study, we examined how varying concentrations of tannic acid influenced larval mosquito survival and found that the removal of microbial communities further reduced larval survivorship, suggesting a protective role of the microbiota. We also employed the next-generation sequencing methods to characterize shifts in microbial communities in both larval habitats and mosquito midguts following tannic acid exposure. Our results support the potential application of tannic acid as a standalone or synergistic agent in mosquito control strategies. Furthermore, a deeper understanding of detoxification mechanisms in *Aedes* mosquitoes and their associated microbiota may reveal novel targets for disrupting vector populations and reducing disease transmission.

## Materials and Methods

### Mosquitoes and experimental design

*Ae. triseriatus* mosquito larvae were from a laboratory colony originating from larvae collected from bark-lined, pan tree holes in American beech (*Fagus grandifolia* Ehr.) on the Michigan State University campus and maintained under standard insectary conditions (27 °C, 12:12 h light:dark cycle, and 85% relative humidity) [29]. Upon adult emergence, mosquitoes were provided with a 10% sucrose solution ad libitum via a cotton wick. Adult *Ae. triseriatus* were blood-fed using bovine blood (Hemostat Lab, Dixon, CA, USA) delivered through an artificial membrane feeder. Two days post-blood meal, oviposition substrates (filter paper moistened with water and placed in Petri dishes) were introduced into rearing cages. Collected eggs were transferred to plastic containers filled with distilled water for hatching. First instar larvae were fed Tetramin tropical fish food flakes (Tetra, Blacksburg, VA, USA) ad libitum, followed by daily ad libitum feedings of Purina Cat Chow (Nestlé) for subsequent instars.

To evaluate the effects of tannic acid (Sigma-Aldrich, St Louis, MO) on mosquito development and microbiome, experimental microcosms were prepared with 1 g of senescent, American beech leaf litter sampled in autumn, dried, and placed into 80 mL of Milli-Q water, and 1 ml of microbial inoculum, consistent with our previous experiments [29,30]. Freshly collected, senescent beech leaves leach up to 8 mg of tannic acid equivalents per gram of dried leaf within the first 24 hours of immersion in water, thus establishing a background of tannin in the microcosm water [31]. Immersed leaves condition rapidly with microbial colonization [30,32]. To evaluate the dose-dependent effect of tannic acid, larvae were hatched from eggs and seeded into microcosms; ten second instar larvae were exposed to a range of TA concentrations (0.00, 0.19, 0.35, 0.72, 1.4, and 1.8 mg/mL). The number of surviving larvae was counted after 1 week. The effects of tannic acid and kanamycin on *Ae. triseriatus* larval survival were assessed by counting surviving larvae one week after treatment [31]. For these combinatorial treatments, four groups were established: (1) kanamycin-only group (100 μg/mL), (2) tannic acid-only group (0.35 mg/mL), (3) tannic acid + kanamycin group (0.35 mg/mL tannic acid + 100 μg/mL kanamycin), and (4) untreated control group. After six days of conditioning, microcosms received either 2 mL tannic acid solution plus 2 mL Milli-Q water (treatment) or 4 mL Milli-Q water only (control). Ten second instar *Ae. triseriatus* larvae were introduced into each microcosm at the time of treatment. Larval survival rate was recorded after seven days of exposure. The experiment was replicated five times, and the data were analyzed using one-way analysis of variance (ANOVA).

### DNA Extraction, Library Construction, and 16S rRNA Sequencing

A subset of microcosms was randomly selected for microbial community analysis after a ten-day incubation. Fourth-instar larvae were collected for sampling. Upon adult emergence, mosquito midguts were immediately dissected and pooled. Before dissection, adults were surface sterilized by three rinses in 70% ethanol followed by one rinse in sterile water. Midguts were removed under sterile conditions using finely sharpened, number 5 watchmaker’s forceps (Daigger Scientific, New Jersey, USA), placed into 200 μL of sterile phosphate-buffered saline (PBS), and pooled from six individuals per sample. Each pooled set was homogenized with a sterile pestle. All dissections and DNA extractions were conducted in a laminar flow biosafety cabinet to avoid contamination. Water samples were collected by centrifuging at 15,000 rpm for 10 min at 4 °C; resulting pellets were stored at −70 °C until further use. Leaf materials after removing surface water were flash-frozen in liquid nitrogen, and ground with a sterile pestle. For larvae, at least 12 replicates were analyzed in the TA-treated group and 10 in the control group. For adults, 14 TA-treated and 19 control samples were processed. For water and leaf samples, five replicates were included for each treatment group.

Mosquito midgut tissues, pellets from water and leaf materials were resuspended in 200 μL of lysis buffer, and DNA was extracted using the DNeasy Blood & Tissue Kit (Qiagen) according to the manufacturer’s protocol. DNA concentration was quantified using Qubit^™^ dsDNA HS Assay Kits, and integrity was confirmed by PCR with primers 63F (CAGGCCTAACACATGCAAGTC) and 1387R (CGGAACATGTGWGGCGGG). Amplicon tagging and 16S rRNA gene sequencing were performed at the Research Technology Support Facility (RTSF) at Michigan State University. The V4 region of the bacterial 16S rRNA gene was amplified using primers 515f (GTG CCA GCM GCC GCG GTA A) and 806r (NNN NNN GGA CTA CHV GGG TWT CTA AT), with unique 6-bp error-correcting barcodes included in the reverse primer. Amplicons were purified, pooled, and sequenced using the Illumina MiSeq platform with a 2 × 250 bp paired-end format and 500-cycle v2 reagent kit. Base calling was performed with Illumina Real Time Analysis (RTA) v1.18.54, and output files were demultiplexed and converted to FASTQ format using Illumina Bcl2fastq v1.8.4.2.3.

### Bioinformatics analysis and statistical analysis

To obtain high-quality sequences, bases at the head or tail with Phred quality scores below Q30 were trimmed, and sequences shorter than 100 bp were discarded. Raw sequence data were processed in QIIME2 (version 2024.2) [33], including sequencing and PCR error reduction, and denoising with DADA2 (filtering, dereplication, chimera removal, and merging of paired-end reads) to generate amplicon sequence variants (ASVs) for taxonomic analysis. ASVs representing less than 0.1% of the total sequences across all samples or annotated as chloroplast or mitochondrial contaminants were removed. Sequencing depth was normalized to the minimum read count among samples. Taxonomic classification was performed using a Naive Bayes classifier trained on the SILVA 138/16S rRNA database (https://www.arb-silva.de/). Alpha diversity was calculated in Mothur (version 1.30.2) (https://mothur.org/) using the Chao1 and Shannon indices based on randomly subsampled sequences from each sample. Beta diversity was assessed using PERMANOVA (999 permutations) based on Bray–Curtis distances, weighted UniFrac, and Jaccard dissimilarities, implemented in the vegan package (version 2.4–1) in R (version 3.3.1). Microbial biomarkers for each experimental group were identified using LEfSe (version 1.0) [34].

Microbial functions were predicted using PICRUSt2 (v2.2.0-b) [35], and functional differences between treatment groups were assessed using FAPROTAX (version 1.2.11) [36]. Predicted genes were annotated against the Enzyme Commission (EC) database (https://enzyme.expasy.org/), the Kyoto Encyclopedia of Genes and Genomes (KEGG) database [37–39], and the MetaCyc Metabolic Pathway Database (https://metacyc.org/). Group differences were analyzed in STAMP, with two-group comparisons performed using the Wilcoxon test followed by false discovery rate (FDR) correction. Statistical significance was defined as P < 0.05.

### Sequence Data Accession Number

Raw paired-end reads per sample without barcode and primer bases were submitted to the Sequence Read Archive of the NCBI (PRJNA1320966).

## Results

### Effects of tannic acid and antibiotic treatment on larval mosquito

**Figure 1.**
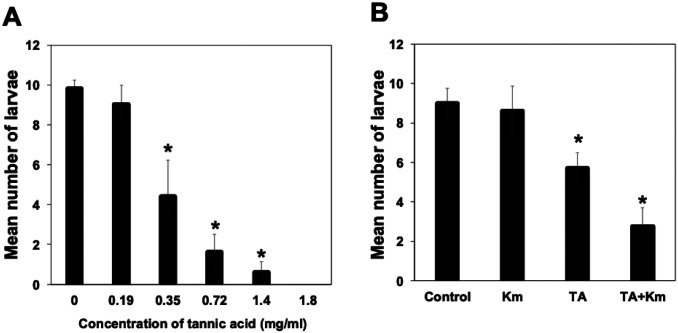
Impact of tannic acid and antibiotic treatment on mosquito larval survival. (A) Survival of *Ae. triseriatus* larvae decreased with increasing TA concentrations. Larvae at the 2^nd^ instar stage were exposed to TA, and surviving individuals were counted after one week. (B) Km treatment reduced microbial protection against TA toxicity. Larvae at the 2^nd^ instar stage were reared under four conditions: control (no TA or antibiotics), TA alone (0.35 mg/ml), Km alone (100 μg/ml), and TA + Km. Km alone had no effect on survival.

Larval *Ae. triseriatus* survival declined with increasing tannic acid concentrations, with 100% mortality observed at 1.8 mg/mL TA (Figure 1A) (ANOVA: F-test, df=4, P < 0.05). Larval survival was high and equivalent between controls and kanamycin alone but was lower with TA (at 35 mg/mL) and lowest with TA and kanamycin in combination (Figure 1B) (ANOVA: F-test, df=4, P < 0.05), indicating a synergistic negative effect on larval survivorship.

### Effects of tannic acid treatment on bacterial composition in insect guts and microcosms

After filtering, denoising, merging, and chimera removal, 3,313,743 high-quality reads were obtained, with a median depth of 44,183 reads per sample. To minimize sequencing depth bias, read counts were normalized to the smallest sample size (1,042 reads) by random subsampling. Rarefaction curves (Figure S1) indicated that samples from rearing water, leaf surfaces, and mosquito midguts (larvae and adults) with or without TA addition did not fully reach asymptotic saturation, suggesting the presence of additional rare taxa. Nonetheless, all curves displayed the characteristic concave upward trend with increasing sequencing effort.

**Figure 2.**
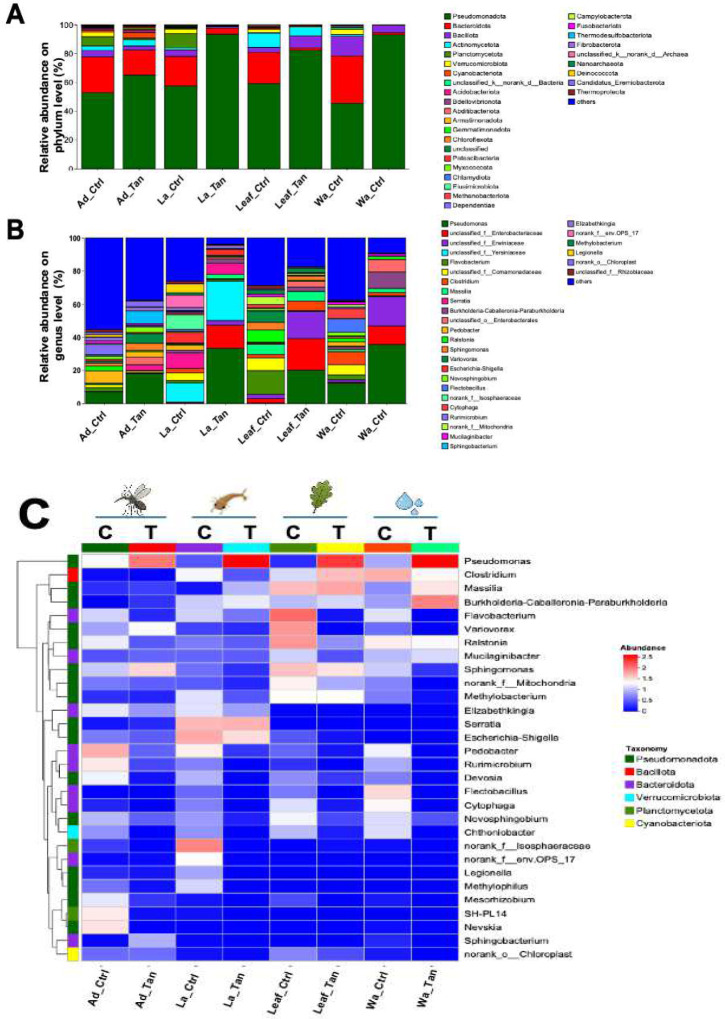
Relative abundance of bacterial communities in microcosms and mosquito midguts with and without TA treatment. (A) Bacterial community composition at the phylum level. (B) Community composition at the genus level. (C) Heatmap of predominant genera across samples. C indicates control (no TA) and T indicates TA treatment. Ad_Ctr, adult mosquitoes without TA; Ad_Tan, adult mosquitoes with TA; La_Ctr, larval mosquitoes without TA; Lar_Tan, larval mosquitoes with TA; Leaf_Ctr, leaf without TA; Leaf_Tan, leaf with TA; Wa_Ctr, water without TA; Wa_Tan, water with TA.

Taxonomic profiling assigned sequences to at least 38 bacterial phyla. The five most abundant were Pseudomonadota, Bacteroidota, Bacillota, Planctomycetota, and Actinomycetota (Figure 2A). Across all habitats (rearing water, leaf surfaces, larval midguts, and adult midguts) Pseudomonadota predominated (45.4–93.5%), regardless of TA treatment. Bacteroidota ranked second (1.7–33.0%). TA addition doubled the relative abundance of Pseudomonadota while sharply reducing Bacteroidota (from 33% to 1.5%), indicating strong TA-mediated inhibition of this bacterial group. Bacillota abundance declined in TA-treated water, Verrucomicrobia nearly disappeared, and Actinomycetota decreased. Similar patterns were observed on leaves and in midguts, except Bacillota increased on TA-treated leaves. Planctomycetota were more common in larvae and adults from non-TA treatments but remained rare in water and leaves.

Genus-level composition varied widely between treatments and sample types (Figure 2B). In control water, ~37.5% of reads were unclassified at the genus level (“others”); dominant genera included *Pseudomonas*, *Flectobacillus*, *Clostridium*, unclassified Comamonadaceae, and *Cytophaga*. TA-treated water showed a marked shift. *Pseudomonas* nearly doubled in abundance (35.6%) while *Flectobacillus*, *Clostridium*, and *Cytophaga* declined to negligible levels (Figure 2C). Conversely, unclassified Erwinaceae, unclassified Enterobacteriaceae, *Burkholderia–Caballeronia–Paraburkholderia*, and unclassified Enterobacteriales were enriched (Figure 2C). In control leaves, *Pseudomonas* was rare (0.4%); dominant genera included *Flavobacterium*, unclassified Comamonadaceae, unclassified Enterobacteriales, *Ralstonia*, and *Variovorax*. TA-treated leaves were dominated by *Pseudomonas*, unclassified Erwinaceae, and unclassified Enterobacteriaceae, resembling TA-treated water communities. In control larval midguts, 55.4% of reads were unclassified (“others”), with *Pedobacter*, *Pseudomonas*, *Rurimicrobium*, *Elizabethkingia*, and *Ralstonia* as key taxa. TA treatment doubled *Pseudomonas* abundance, while *Elizabethkingia* and *Pedobacter* remained similar to controls; *Sphingobacterium*, *Serratia*, and *Variovorax* were also common (Figure 2C). Adult midguts in controls were dominated by unclassified Yersiniaceae, *Serratia*, unclassified Isosphaeraceae, unclassified env.OPS_17, *Escherichia–Shigella*, and *Legionella*. In TA-treated adults, *Pseudomonas*, unclassified Enterobacteriales, and unclassified Yersiniaceae increased markedly (33.3%, 23.8%, and 14.0%, respectively).

**Figure 3.**
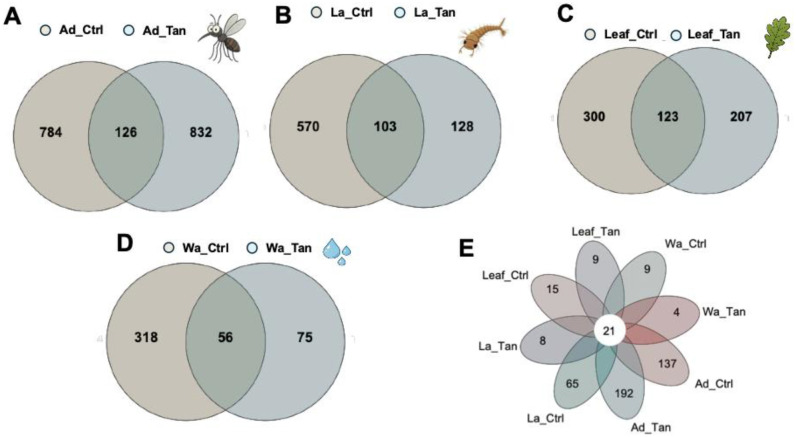
Venn diagrams of shared and unique ASVs among TA-treated and control samples. The diagrams illustrate the overlap of ASVs between TA-treated and control groups for adult mosquitoes, larval mosquitoes, water, and leaf surfaces. Ad_Ctr, adult mosquitoes without TA; Ad_Tan, adult mosquitoes with TA; La_Ctr, larval mosquitoes without TA; Lar_Tan, larval mosquitoes with TA; Leaf_Ctr, leaf without TA; Leaf_Tan, leaf with TA; Wa_Ctr, water without TA; Wa_Tan, water with TA.

Venn analysis showed that TA-treated and control samples shared only a limited number of ASVs (Figure 3). In adult guts, 1,742 ASVs were detected - 126 (7.2%) shared, 832 (47.8%) unique to TA-treated, and 784 (45.0%) unique to controls (Figure 3A). In larval guts, 801 ASVs were identified - 103 (12.9%) shared, 570 (71.2%) unique to controls, and 128 (16.0%) unique to TA-treated (Figure 3B). Leaves had 630 ASVs - 123 (19.5%) shared, 300 (47.6%) control-specific, and 207 (32.9%) TA-specific (Figure 3C). Water had 449 ASVs - 56 (12.5%) shared, 318 (70.8%) control-specific, and 75 (16.7%) TA-specific (Figure 3D). Across all habitats combined, only 21 ASVs (4.8%) were shared (Figure 3E).

### Alpha and beta diversity affected by TA in the insect guts and microcosms

**Figure 4.**
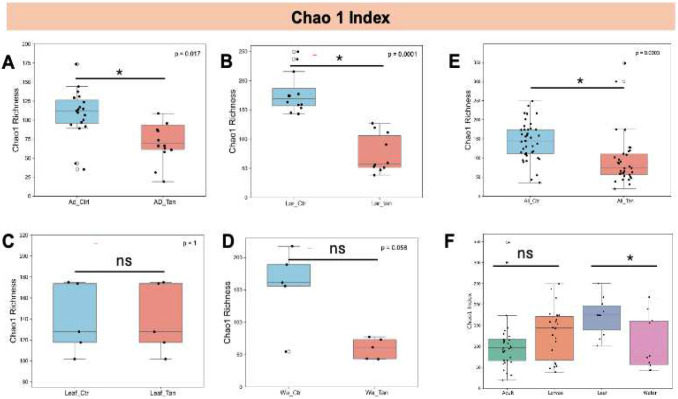
Chao1 richness of gut microbiota across *Aedes* mosquito developmental stages and environmental microcosms. (A) Adults with and without TA treatment. (B) Larvae with and without TA treatment. (C) Leaf samples with and without TA treatment. (D) Water samples with and without TA treatment. (E) Combined samples (adults, larvae, leaves, water) with and without TA treatment. (F) Comparison across adults, larvae, leaves, and water, regardless of TA treatment. Diversity metrics are based on total ASVs. Statistical differences were assessed using the Kruskal–Wallis test. * indicates p < 0.05; Ns, not significant (p > 0.05). Ad_Ctr, adult mosquitoes without TA; Ad_Tan, adult mosquitoes with TA; La_Ctr, larval mosquitoes without TA; Lar_Tan, larval mosquitoes with TA; Leaf_Ctr, leaves without TA; Leaf_Tan, leaves with TA; Wa_Ctr, water without TA; Wa_Tan, water with TA.

Building on our initial observations of microbial community composition, we next assessed whether alpha diversity was influenced by TA treatment (Figure 4). Bacterial richness, as measured by the Chao1 index, was significantly reduced in TA-treated adult guts compared to controls (p < 0.05) (Figure 4A). A similar decrease in richness was observed in larval samples following TA exposure (Figure 4B). In contrast, no significant richness differences were detected between TA-treated and untreated leaf samples (p > 0.05) (Figure 4C), nor in water samples (Figure 4D). However, when comparing all non-TA-treated samples (water, leaf, larvae, and adult grouped as controls) against all TA-treated samples (Figure 4E), a significant difference emerged (p < 0.05). Additionally, leaf samples generally exhibited higher Chao1 values than water samples, independent of treatment. When TA treatment was disregarded, no significant differences in richness were found between treated and untreated adults or larvae (p > 0.05) (Figure 4F).

**Figure 5.**
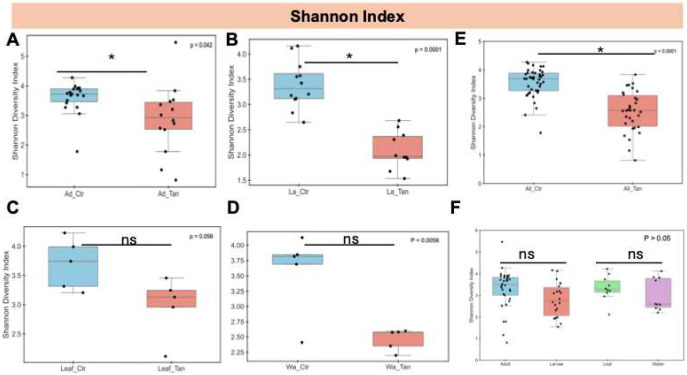
Shannon diversity index of gut microbiota across *Aedes* mosquito developmental stages and environmental microcosms. (A) Adults with and without TA treatment. (B) Larvae with and without TA treatment. (C) Leaf surfaces with and without TA treatment. (D) Water samples with and without TA treatment. (E) Combined samples (adults, larvae, leaves, water) with and without TA treatment. (F) Comparison across adults, larvae, leaves, and water, regardless of TA treatment. Diversity metrics are based on total ASVs. Statistical differences were assessed using the Kruskal–Wallis test. * indicates p < 0.05; ns, not significant (p > 0.05). Ad_Ctr, adult mosquitoes without TA; Ad_Tan, adult mosquitoes with TA; La_Ctr, larval mosquitoes without TA; Lar_Tan, larval mosquitoes with TA; Leaf_Ctr, leaves without TA; Leaf_Tan, leaves with TA; Wa_Ctr, water without TA; Wa_Tan, water with TA.

Significant differences in species diversity, assessed via the Shannon index, were found between non-TA-treated controls and TA-treated adults (p < 0.05), with TA-treated larvae also showing reduced diversity relative to controls (Figure 5A and 5B). However, no significant diversity differences (p > 0.05) were observed between TA-treated and control leaves, nor between TA-treated and control water samples (Figure 5C and 5D). When ignoring TA treatment, Shannon diversity did not differ significantly (p > 0.05) among adult and larval mosquitoes or between leaf and water samples (Figure 5E and 5F).

**Figure 6.**
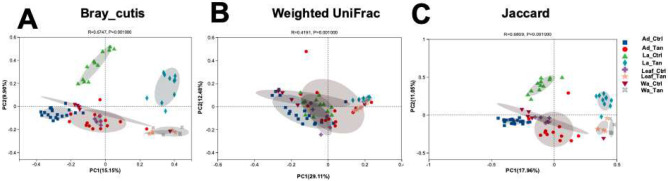
Principal coordinate analysis (PCoA) of bacterial community structure. (A) Bray–Curtis distances. (B) Weighted UniFrac distances. (C) Jaccard distances. Distances between symbols on each ordination plot indicate relative dissimilarities in community composition. Ad_Ctr, adult mosquitoes without TA; Ad_Tan, adult mosquitoes with TA; La_Ctr, larval mosquitoes without TA; Lar_Tan, larval mosquitoes with TA; Leaf_Ctr, leaves without TA; Leaf_Tan, leaves with TA; Wa_Ctr, water without TA; Wa_Tan, water with TA.

For beta diversity analysis, we performed PCoA to assess similarities in microbial structures among TA treatment, rearing systems and development stages. PCoA analyses were based on Bray-Curtis distance, Weighted UniFrac and Jaccard, respectively (Figure 6). For Bray-Curtis distance analysis (Figure 6A), the dissimilarity revealed clear separation between TA-treated and control microbial communities (ANOSIM: R = 0.67, p = 0.001). The first two PCoA axes explained 25.05% of the total variance (PCoA1: 15.15%, PCoA2: 9.90%). Samples clustered primarily by TA treatment, with separation along axis 1 except for adult mosquito samples. Moreover, both Weighted UniFrac (ANOSIM: R = 0.42, p = 0.001) and Jaccard (ANOSIM: R = 0.68, p = 0.001) distance analyses supported significant structural differences between TA-treated and control microbiome (Figure 6B and 6C), agreeing with Bray-Curtis analysis. From the three different dissimilarity analyses, we also observed there were distinct gut microbial communities across developmental stages (larvae vs. adults) and in rearing systems (leaf vs. water) (Figure 6). Moreover, PERMANOVA analysis indicated that TA treatment (P = 0.001), rearing systems (P = 0.028), and developmental stages (P = 0.001) were all significant drivers of beta diversity (Table 1).

### Linear discriminant analysis effect size (LEfSe) analysis

**Figure 7.**
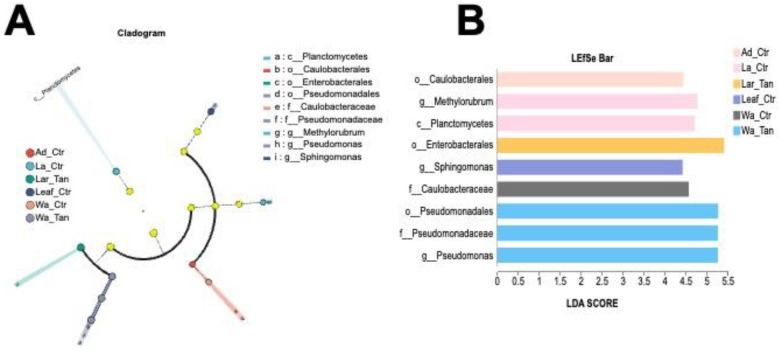
LEfSe analysis showing significant differences of microbial species from phylum to genus. A) Cladogram indicates the phylogenetic distribution of microbial communities in insect guts and rearing system and their TA-treated samples. Yellow nodes represent microbial taxa with no significant difference between different life stages, while other color nodes represent microbial taxa that are significantly enriched at those life stages. B) LEfSe Bar diagram for different samples with LDA scores higher than 2.0. A higher LDA score represents that this bacterial taxon has a greater contribution to the differences.

LEfSe analysis identified bacterial taxa differentially enriched across mosquito life stages and microcosms, spanning from phylum to genus level (Figure 7A and 7B). Only taxa with an LDA score greater than 2.0 were included. The cladogram revealed distinct phylogenetic patterns, highlighting three candidate markers in TA-treated water: order Pseudomonadales, family Pseudomonadaceae, and genus *Pseudomonas*. In contrast, family Caulobacteraceae was enriched exclusively in control adults, representing non-treated water. Two markers such as genus *Methylorubrum* and class Planctomycetes were enriched in control larvae, while genus *Sphingomonas* was a marker for TA-treated larvae. Additionally, family Caulobacteraceae served as a characteristic marker in leaf-reared control adults. No marker taxa were detected in treated leaf or treated adult groups.

### Functional prediction analysis reveals potential differences in microbial community metabolic function.

**Figure 8.**
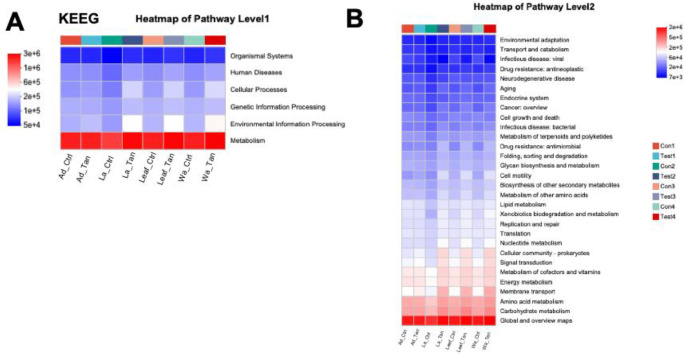
Prediction of KEGG functions [37–39] of bacteria at different developmental stages, with TA-treatment and in the microcosms. A) Level 1 of predicted function categories is shown in each group. B) Level 2 of predicted function categories is shown in each group. Ad_Ctr, adult mosquitoes without tannic acid treatment; Ad_Tan, adult mosquitoes with tannic acid treatment; La_Ctr, larval mosquitoes without tannic acid treatment; Lar_Tan, larval mosquitoes with tannic acid treatment; Leaf_Ctr, leaf without tannic acid treatment; Leaf_Tan, leaf with tannic acid treatment; Wa_Ctr, water without tannic acid treatment; Wa_Tan, water with tannic acid treatment.

**Figure 9.**
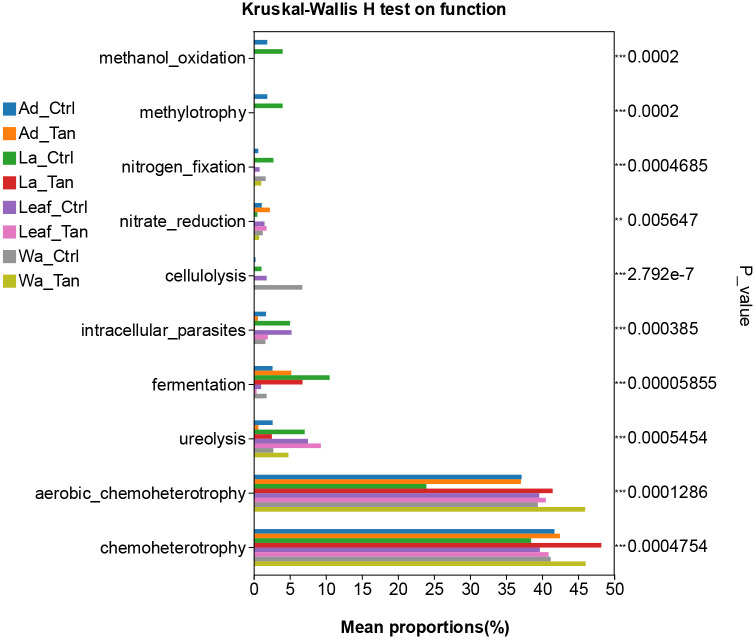
Functional differences among all groups. The relative proportions of FAPROTAX-predicted functions were compared across mosquito developmental stages, TA treatments, and microcosm samples. p-values are shown along the right Y-axis to indicate significant differences among the selected groups.

**Figure 10.**
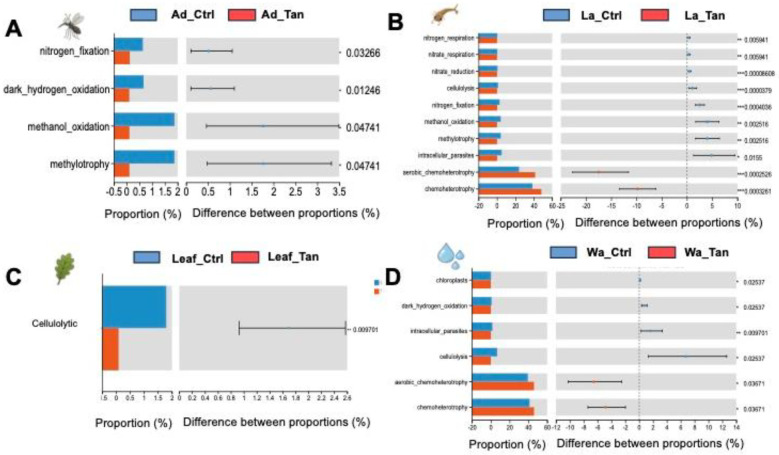
Paired comparation of Functional differences. The relative proportions of FAPROTAX-predicted functions were compared. (A) Adults with and without TA treatment. (B) Larvae with and without TA treatment. (C) Leaf surfaces with and without TA treatment. (D) Water samples with and without TA treatment.

PICRUSt2 predicted six major functional pathways (Level 1) based on KEGG (Figure 8A). These functional categories were enriched in metabolism, environmental information processing, genetic information processing, cellular processes, human diseases, and organismal systems (Figure 8A). Among all groups, metabolism was the predominant predicted function (72.91–76.5%), followed by environmental information processing (6.07–9.55%) and genetic information processing (4.80–6.50%). It is noted that more “environmental information processing” functions were predicted in the TA-treated samples. The most abundant pathways at Level 2 included carbohydrate metabolism, global and overview maps, amino acid metabolism, energy metabolism, and metabolism of cofactors and vitamins (Figure 8B). However, COG functional classification did not clearly resolve the predicted functional differences (Figure S2). Therefore, FAPROTAX was employed to refine functional predictions, focusing on processes such as plant cell wall degradation, nitrogen and carbon cycling, and nutrient assimilation, based on individual microbial profiles. This annotation identified 30 functional categories within the insect guts and rearing systems (supplemental Figure 3). The top five enriched pathways were chemoheterotrophy, aerobic chemoheterotrophy, fermentation, and ureolysis (Figure S3). We further analyzed the different predicted function enriched in treatment for further comparation (Figure 9 and Figure 10). Notably, cellulolysis and intracellular parasite-related functions were more abundant in water controls without TA compared to TA-treated water (Figure 9). The only significantly different pathway between TA-treated and untreated leaf surface samples was cellulolytic activity (Figure 10C). Furthermore, aromatic compound degradation and nitrate ammonification pathways were more prominent in water, leaf, and larval samples without TA. Conversely, “methanol oxidation” and “methylotrophy” were more abundant in larval and adult mosquitoes without TA, which likely benefits insect physiology (Figure 10A ang 10B). Genes involved in nitrogen metabolism were commonly detected in both larval and adult mosquitoes (Figure 10A and Figure 10B). Overall, TA-treated insects showed a reduced proportion of nitrogen fixation and methanol metabolism genes. However, several functions such as aerobic chemoheterotrophy showed higher abundance in TA-treated water and larval samples (Figure 10B and Figure 10D).

## Discussion

Previous research on tannin-insect interactions largely focused on their impacts on the growth and physiology of herbivorous pests, with relatively little attention paid to aquatic insects [8,18,21,22]. In this study, we investigated the effects of TA on mosquito larval development, gut microbiota composition, and environmental microbial communities within experimental rearing microcosms. Our research centered on the feeding ecology of *Ae. triseriatus* larvae in container habitats, where early-life interactions with microbial communities can influence adult fitness and vector competence [40–43]. Larval *Ae. triseriatus* feed by filtering and browsing suspended particulate matter, detritus of plant leaves, and microbial biofilms found on submerged surfaces such as plant material and container walls [43]. In natural tree holes, microbial community composition was shaped by both leaf litter decomposition and larval mosquito activity [44]. However, little is known about how mosquito larvae tolerate or metabolize plant-derived polyphenol compounds such as tannins, which commonly accumulate in these environments [7]. The western tree hole mosquito was negatively affected by exposure to tannins, but any ameliorating effects of microorganisms on the effects were not investigated [5,6]. Overall, our findings demonstrated tannic acid influenced both mosquito survival and the structure of their associated microbial communities. The response of microbiota to elevated tannin acid concentrations suggested a potential microbial role in detoxification processes. These insights expand our understanding of how ecological and chemical factors shape mosquito–microbiome interactions and suggest that naturally occurring compounds such as tannins could be leveraged to manipulate mosquito-associated microbiota as part of integrated biocontrol strategies against mosquito populations and vector-borne diseases [18,21].

Our results showed that elevated TA concentrations significantly reduced larval survival rate. Although TA likely exerts its effects primarily by directly damaging the midgut epithelium of *Aedes* mosquitoes as reported previously [5,7], we cannot rule out additional pathways through which TA may influence larval physiology [11,18]. For example, tannic acid (TA) was shown to increase reactive oxygen species while decreasing antioxidant and detoxification enzyme activities in hosts [18]. In addition, dietary tannins were reported to change growth, behavior, and the gut microbiome of larval amphibians [45]. Because larval *Aedes* mosquitoes depend on microbial and detrital food resources that are frequently coated with plant-derived tannins, they are continuously exposed to these chemical stressors [30,32,43]. Such exposure may lower the nutritional quality of available food, disrupt gut microbial communities, and impose sublethal physiological costs that collectively slow larval growth and development. Another possible pathway for tannic acid–induced toxicity involves changes in water chemistry, especially reductions in dissolved oxygen (DO) levels, which may result from increased microbial respiration during the breakdown of tannin-rich organic matter [46]. Soaking American beech leaves for three days released tannins up to 120 mg/L, substantially lowering DO [46]. Reduced DO imposed metabolic stress, increased respiration costs, and impaired larval growth and survival [47]. Whether these mechanisms apply to mosquito larvae and other aquatic insects remains to be determined. Taken together, findings in this study and others indicate that tannins possibly affect mosquito survival through multiple, interacting pathways including direct physiological stress, altered food quality, gut microbiome disruption, and changes in water chemistry. It highlights their potential as natural compounds to modulate mosquito populations and inform integrated vector management strategies.

Exogenous toxicants are known to perturb insect gut microbiota, disrupting physiological homeostasis and impairing host growth and development [48]. Consistent with this, TA exposure significantly altered the richness, diversity, and taxonomic composition of gut microbiota in both larval rearing systems and the insects themselves, with TA-treated groups exhibiting reduced Chao1 richness and lower Shannon diversity indices compared to controls. PCoA analysis revealed clear differences in microbial community structures, highlighting a strong TA-driven influence on community composition. These shifts suggested that TA exposure selectively favored microbes capable of detoxification or otherwise supporting host health under chemical stress. Despite its antimicrobial properties, many bacteria exhibit tannin resistance [26,49–51]. Microbes have evolved diverse mechanisms for TA degradation in natural environments [51,52]. Notably, the relative abundance of *Pseudomonas* spp. consistently increased across insect midguts, rearing water, and leaf surfaces under sublethal TA exposure compared to controls. In agreement with this observation, Tan et al. reported a higher relative abundance of *Pseudomonas* in TA-treated larvae compared to untreated controls (37.43% vs. 33.59%) [18]. In *Pseudomonas protegens* Pf-5, TA exposure modulated iron homeostasis, upregulating genes for heme uptake and siderophore biosynthesis and transport, which may reduce reactive oxygen species generation and enhance bacterial survival [53]. Additionally, certain *Pseudomonas* species can metabolize tannic acid and its hydrolysis product gallic acid as sole carbon sources [54]. TA’s antimicrobial and protein-binding properties may inhibit tannin-sensitive bacteria, reducing competition and selectively enriching for tannin-tolerant taxa like *Pseudomona*s [50]. TA may alter the microenvironment within mosquito larval guts or associated habitats (e.g., pH, nutrient availability), indirectly favoring *Pseudomonas* proliferation [45]. As predominant mosquito commensals, *Pseudomonas* spp. played key roles in shaping larval gut communities and protecting hosts from toxic metals and insecticides [55], with disruptions to these populations linked to increased larval mortality and impaired development [56]. Collectively, these findings suggest that TA exposure drives a selective restructuring of mosquito gut microbiota, promoting tannin-tolerant taxa like *Pseudomonas*, which may provide detoxification functions and confer mutualistic benefits to the host.

Microbes within *Burkholderia–Caballeronia-Paraburkholderia*, unclassified Erwinaceae, and unclassified Enterobacteriaceae were also promoted by TA addition (Figure 2). Members of the Erwinaceae family, such as *Pantoea* and *Erwinia*, were commonly plant-associated bacteria [57,58]. Many plant-associated bacteria possessed tannin-tolerant or tannin-degrading enzymes including tannase (tannin acyl hydrolase) and polyphenol oxidases to survive in these phenolic-rich environments [27,51]. For instance, *P. agglomerans* was reported to tolerate polyphenols and degrade certain gallotannins [59]. *Burkholderia* exhibited a dramatic increase in relative abundance in water samples treated with TA compared to controls. It was also found in *Aedes* mosquito habitats [40,41]. This marked enrichment suggested a strong tolerance or adaptive response of *Burkholderia* to TA exposure, potentially mediated by specific genetic determinants that confer resistance or degradation capabilities. *Serratia* spp., another common commensal bacterium associated with mosquitoes [60], demonstrated a modest increase in relative abundance in TA-treated larval mosquitoes and persisted in adult mosquitoes. However, the physiological functions and ecological roles of *Serratia* in mosquito biology remain poorly characterized, warranting further investigation. The elevated abundance of *Pseudomonas, Serratia*, and *Burkholderia* collectively implies that these taxa may harbor intrinsic or acquired genes enabling resistance to the antimicrobial and protein-binding effects of TA, thereby facilitating their persistence and proliferation in TA-enriched environments [61,62].

In contrast, TA exposure resulted in a notable decrease in the relative abundance of *Flavobacterium* across both microcosms and mosquitoes [40,41]. The reduction of *Flavobacterium* is particularly significant, as this genus represents an important commensal gut bacterium in mosquitoes, commonly acquired and enriched from larval habitats [42]. Prior studies demonstrated that *Flavobacterium* played critical roles in mosquito nutrition and fitness [42]; its removal delayed larval growth, reduced adult longevity, and impaired overall mosquito fitness [63]. The antimicrobial properties of TA against this group highlight the potential ecological consequences of tannin presence in mosquito breeding sites, possibly influencing microbial community structure and, by extension, host health and development. Understanding these microbial dynamics is essential for elucidating the complex interactions between environmental chemicals, microbiota, and mosquito physiology.

LEfSe analysis identified Pseudomonadaceae and the genus *Pseudomonas* as markers enriched in TA-treated water, highlighting their role in tannic acid degradation or tolerance. In contrast, Caulobacteraceae were enriched only in non-treated water. It features a distinctive life cycle with motile swarmer and sessile stalked cells, enabling effective colonization of aquatic surfaces [64]. Widely distributed in various habitats, Caulobacteraceae contribute to nutrient cycling and organic matter decomposition [64,65], particularly in nutrient-poor environments such as water in native tree hole or disposed tires. In control larvae, two additional markers appeared: genus *Methylorubrum* and class Planctomycetes. Bacteria *Methylorubrum* are facultative methylotrophs that utilize single-carbon compounds like methanol, supporting carbon cycling and potentially host nutrition [66]. Planctomycetes are involved in nitrogen and carbon cycling and may help maintain gut microbial balance [67]. *Sphingomonas* species [68], common in various insect microbiomes including mosquitoes, were markers in TA-treated larvae. Known for degrading aromatic compounds, *Sphingomonas* likely aid in detoxifying tannin-rich environments [69]. They were also found in herbivorous insects, assisting in plant material digestion [70]. Lastly, Caulobacteraceae was a marker in leaf-reared control adults. LEfSe analysis did not discover significant marker taxa in two groups such as TA-treated leaf or treated adult groups. This usually means that those groups don’t have bacterial taxa with strong enough differential abundance (LDA score > 2.0) compared to others, or the microbial communities are more similar in these groups.

Functional predictions using PICRUSt2 revealed that carbohydrate and amino acid metabolism dominated microbial activity in mosquito guts and their habitats, while FAPROTAX identified key functions including chemoheterotrophy and fermentation, essential for nutrient cycling and organic matter breakdown. Cellulolytic activity and aromatic compound degradation were higher in untreated water and leaf samples, suggesting that TA inhibits microbes responsible for plant polymer and polyphenol degradation [71]. This shift likely alters microbial community composition and reduces the capacity to process tannin acid. Methanol oxidation and methylotrophy pathways were more abundant in untreated larvae and adults but reduced under TA treatment, potentially impacting host nutrition and metabolite production beneficial for mosquito development. Conversely, aerobic chemoheterotrophy increased in TA-treated water and larvae, indicating enrichment of microbes capable of tolerating or metabolizing tannic acid. Nitrogen metabolism genes were present across samples, but nitrogen fixation decreased in TA-treated insects, potentially limiting nitrogen availability critical for larval growth. Overall, TA reshaped mosquito-associated microbial functions by suppressing specific degradation pathways while promoting others, thereby influencing microbial detoxification capacity and nutrient support, which likely affected mosquito development and survival in tannin-rich environments.

## Supplementary Material

1

## Figures and Tables

**Table 1:** Pseudo *F* table of pairwise comparisons of TA types using PERMANOVA

Pairwise comparison	Source of variance	Degrees of freedom	Sum of squares	Mean square	*F*	*R*2	*p*
Water*Leaf	Microcosm	1	0.70	0.69	2.20	0.11	**0.028**
	Residuals	18	5.66	0.31		0.89	
	Total	19	6.35				
TA*Control	Treatment	1	0.64	3.05	8.05	0.01	**0.001**
	Residuals	73	27.68	0.34		0.9	
	Total	74	30.73			1	
Adults*Lavae	Development	1	3.22	3.32	9.08	0.15	**0.001**
	Residuals	1	18.77	0.35		0.85	
	Total	54	21.99			1	

## Data Availability

Raw paired-end reads per sample without barcode and primer bases were submitted to the Sequence Read Archive of the NCBI (PRJNA1320966).
