# Supplementary material for: Tannic Acid Shaped Microbiome Composition in Midguts and Rearing Microcosms of Aedes triseriatus (Say)
Source: Res Sq. 2025 Oct 27:rs.3.rs-7706154. Preprint. [Version 1] doi: 10.21203/rs.3.rs-7706154/v1 (PMC12636730; doi:10.21203/rs.3.rs-7706154/v1)
Supplement: 1 [file NIHPPRS7706154V1-supplement-1.pdf]

Supplemental Materials

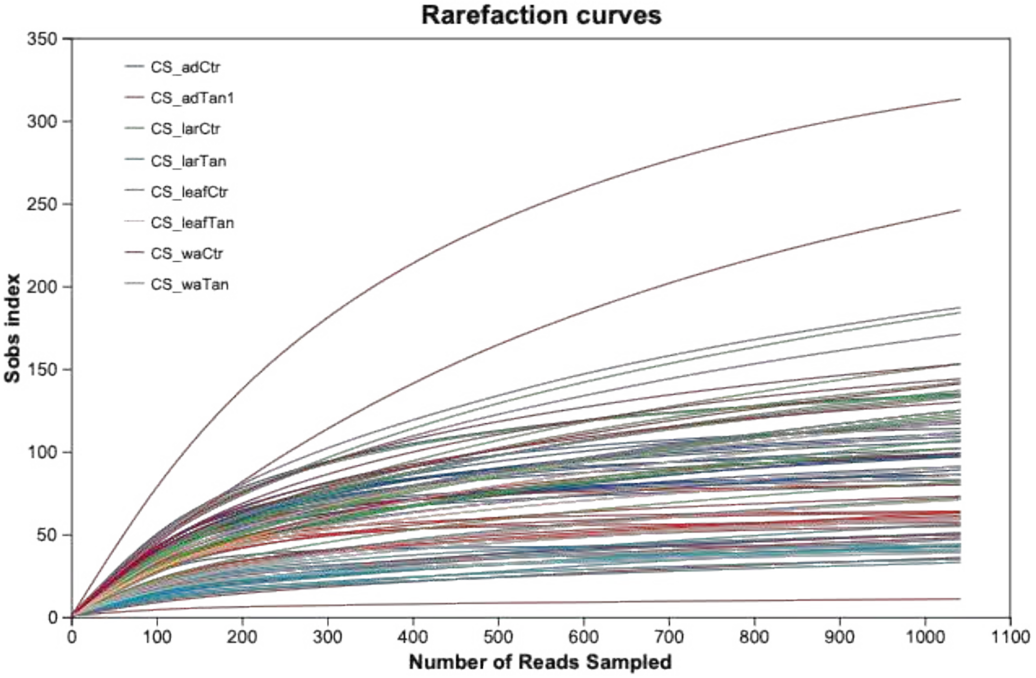

Figure S1 Rarefaction analysis of bacterial 16S rRNA gene libraries from the rearing water, leaf surfaces, larval and adult *A. triseriatus* mosquitoes. Operational taxonomic units (OTUs) were grouped with a 97% similarity. adCtr, adult mosquitoes without tannic acid treatment; adTan, adult mosquitoes with tannic acid treatment; larCtr, larval mosquitoes without tannic acid treatment; larCtr, larval mosquitoes with tannic acid treatment; leafCtr, leaf without tannic acid treatment; leafTan, leaf with tannic acid treatment; waCtr, water without tannic acid treatment; waTan, water with tannic acid treatment.

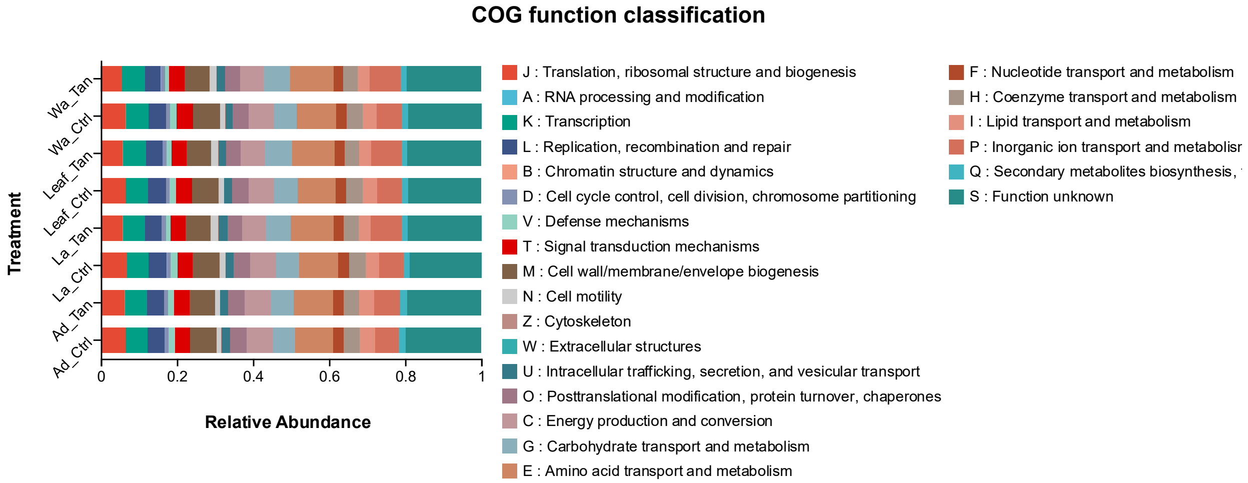

Figure S2 COG function classification of the predicted functions by PICRUSt2 analysis. Ad_Ctr, adult mosquitoes without tannic acid treatment; Ad_Tan, adult mosquitoes with tannic acid treatment; La_Ctr, larval mosquitoes without tannic acid treatment; Lar_Tan, larval mosquitoes with tannic acid treatment; Leaf_Ctr, leaf without tannic acid treatment; Leaf_Tan, leaf with tannic acid treatment; Wa_Ctr, water without tannic acid treatment; Wa_Tan, water with tannic acid treatment.

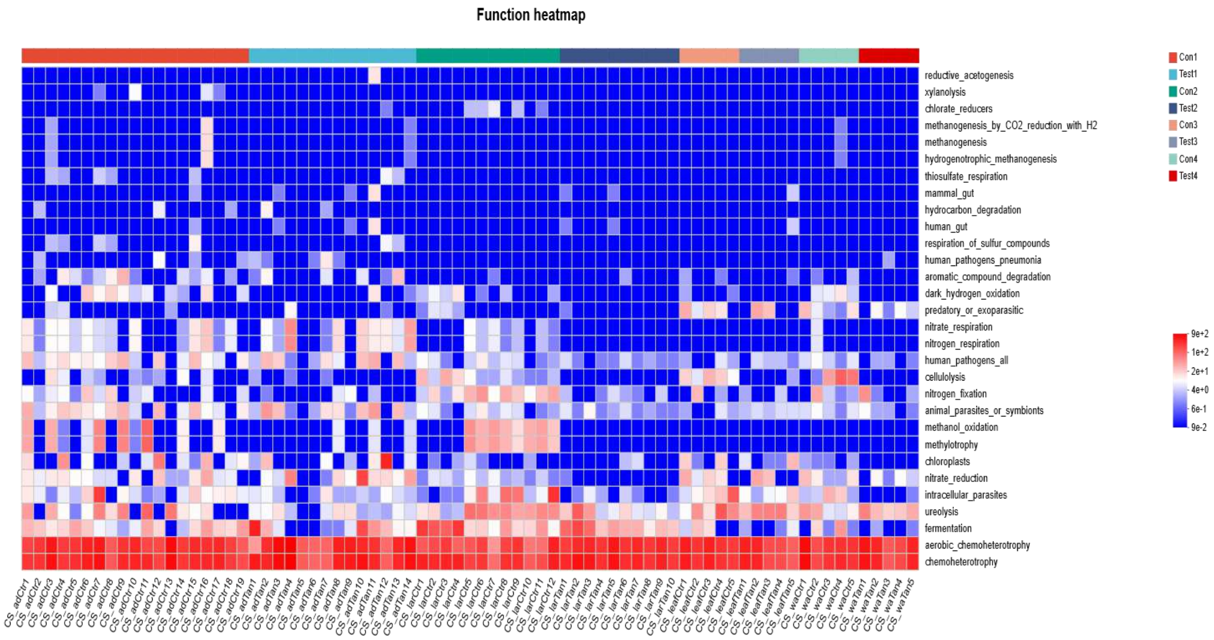

Figure S3 Functional prediction among all groups by FAPROTAX.
